# Supplementary material for: Impact of COVID-19 Pandemic on Initiation of Immunosuppressive Treatment in Immune-Mediated Inflammatory Diseases in Austria: A Nationwide Retrospective Study
Source: J Clin Med. 2022 Sep 9;11(18):5308. doi: 10.3390/jcm11185308 (PMC9503597; doi:10.3390/jcm11185308)
Supplement: Supplementary file 1 [file jcm-11-05308-s001.zip › jcm-1883841-supplementary.pdf]

Journal of
Clinical Medicine

MDPI

Supplementary Material

Article

Impact of COVID-19 Pandemic on Initiation of Immunosuppressive Treatment in Immune-Mediated Inflammatory Diseases in Austria: A Nationwide Retrospective Study

Maximilian Kutschera [1], Valentin Ritschl [2,3], Berthold Reichardt [4], Tanja Stamm [2,3,*], Hans Kiener [5], Harald Maier [6], Walter Reinisch [1], Bernhard Benka [7] and Gottfried Novacek [1]

[1] Department of Internal Medicine III, Division of Gastroenterology and Hepatology, Medical University of Vienna, 1090 Vienna, Austria
[2] Institute of Outcomes Research, Center for Medical Statistics, Informatics and Intelligent Systems, Medical University of Vienna, 1090 Vienna, Austria
[3] Ludwig Boltzmann Institute for Arthritis and Rehabilitation, 1090 Vienna, Austria
[4] Austrian Health Insurance Fund, Eisenstadt, Austria
[5] Department of Internal Medicine III, Division of Rheumatology, Medical University of Vienna, 1090 Vienna, Austria
[6] Department of Dermatology, Medical University of Vienna, 1090 Vienna, Austria
[7] AGES—Austrian Agency for Health and Food Safety Ltd., Division of Public Health, 1220 Vienna, Austria
* Correspondence: tanja.stamm@meduniwien.ac.at; Tel.: +43-1-40400-16370

Citation: Kutschera, M.; Ritschl, V.; Reichardt, B.; Stamm, T.; Kiener, H.; Maier, H.; Reinisch, W.; Benka, B.; Novacek, G. Impact of COVID-19 Pandemic on Initiation of Immunosuppressive Treatment in Immune-Mediated Inflammatory Diseases in Austria: A Nationwide Retrospective Study. *J. Clin. Med.* **2022**, *11*, 5308. https://doi.org/10.3390/jcm11185308

Academic Editors: Francesco Pugliese, Francesco Alessandri and Giovanni Giordano

Received: 9 August 2022
Accepted: 3 September 2022
Published: 9 September 2022

Publisher's Note: MDPI stays neutral with regard to jurisdictional claims in published maps and institutional affiliations.

Copyright: © 2022 by the authors. Submitted for possible open access publication under the terms and conditions of the Creative Commons Attribution (CC BY) license (https://creativecommons.org/licenses/by/4.0/).

Supplemental Table S1. Diagnoses of immune-mediated inflammatory diseases in 56,913 out of 95,573 patients.

Diagnosis	n (%)
Inflammatory bowel disease	41,631 (43.5)
Crohn's disease	3529 (3.7)
Plaque Psoriasis/Psoriatic arthritis	3091 (3.2)
Ulcerative Colitis	2,21 (3.0)
Rheumatoid arthritis	1995 (2.1)
Atopic dermatitis	1710 (1.8)
Plaque Psoriasis	1287 (1.4)
Ankylosing spondylitis	259 (0.3)
Hidradenitis suppurativa	179 (0.2)
Childhood arthritis	152 (0.2)
Uveitis	112 (0.1)
Lupus erythematosus	79 (0.1)
Psoriatic arthritis	35 (0.04)
Behçet-disease	33 (0.04)

We assigned diagnoses based on medications which were only approved for one specific indication during the observation period: Abatacept was only approved for rheumatoid arthritis, apremilast for plaque psoriasis and

psoriasis arthritis (a differentiation between both indications was not possible), belimumab for lupus erythematodes, brodalimumab for plaque psoriasis, dupilumab for atopic dermatitis, filgotinib for rheumatoid arthritis (approval for ulcerative colitis occurred after the observation period), guselkumab for plaque psoriasis and psoriasis arthritis (a differentiation was not possible), mesalazine for inflammatory bowel disease (a differentiation between Crohn's disease and ulcerative colitis was not possible), risankizumab for plaque psoriasis, sarilumab for rheumatoid arthritis, tildrakizumab for plaque psoriasis, and vedolizumab for inflammatory bowel disease (a differentiation between Crohn's disease and ulcerative colitis was not possible). These additional diagnoses were added to the coded diagnoses of hospitalizations (Table 1 of the manuscript). In another 38,860 patients the diagnosis was not available. .

Due to the frequent use of mesalazine (approved for inflammatory bowel diseases), IBD is overrepresented in the data set compared to other immune-mediated inflammatory diseases.

Supplemental Table S2. Starts of medications during the observation period 2017 to 2020 (n=122,213).

Medication	Absolute frequency
Abatacept	1265
Adalimumab	12,867
Anakinra	314
Azathioprine	7661
Baricitinib	1486

Belimumab	170
Brodalumab	197
Canakinumab	16
Certolizumab pegol	1037
Ciclosporin	2239
Dupilumab	2056
Etanercept	4631
Golimumab	2838
Infliximab	2234
Ixekizumab	1835
Leflunomid	4098
Mesalazine	48,402
Methotrexate	87
Mycophenolacid	4920
Risankizumab	410
Rituximab	559
Sarilumab	21
Secukinumab	2321
Sulfasalazine	7415

Tildrakizumab	232
Tocilizumab	3402
Tofacinitib	1585
Upadacitinib	472
Ustekinumab	2486
Vedolizumab	1416

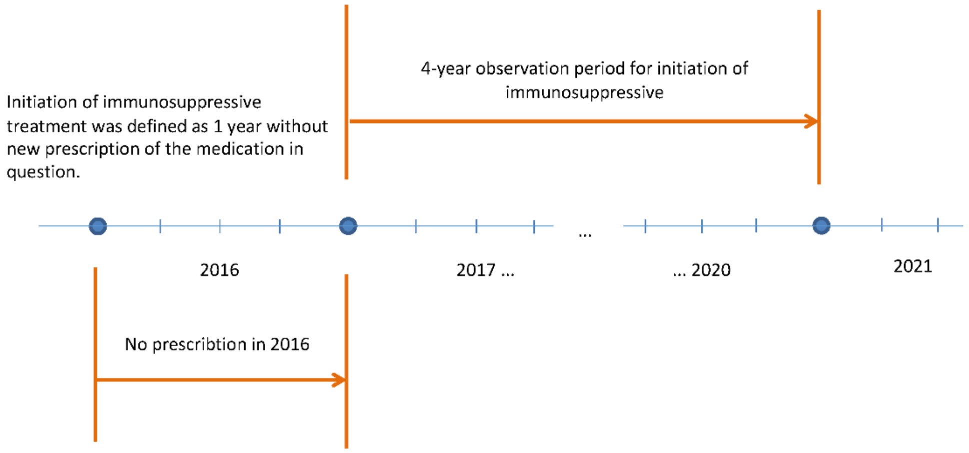

Supplemental Figure S1. Timeline of the study.

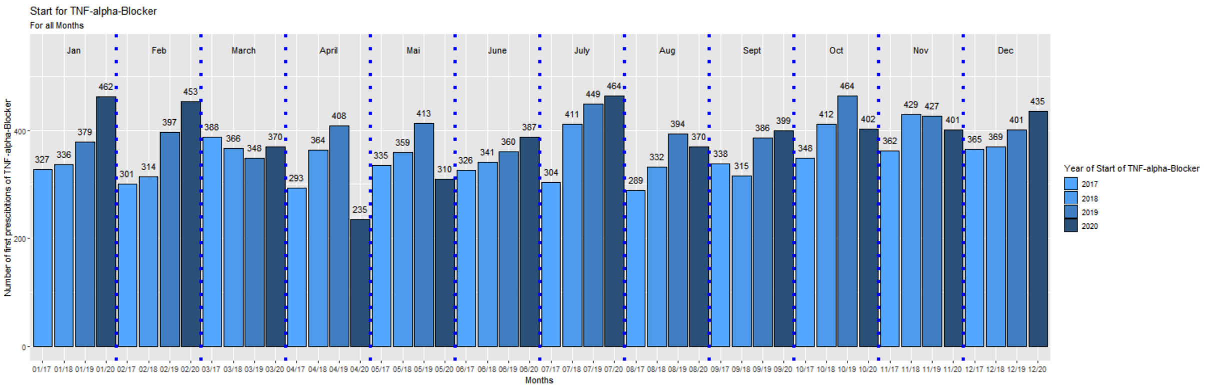

Supplemental Figure S2. Start of TNFα inhibitors in the period 2017-2020

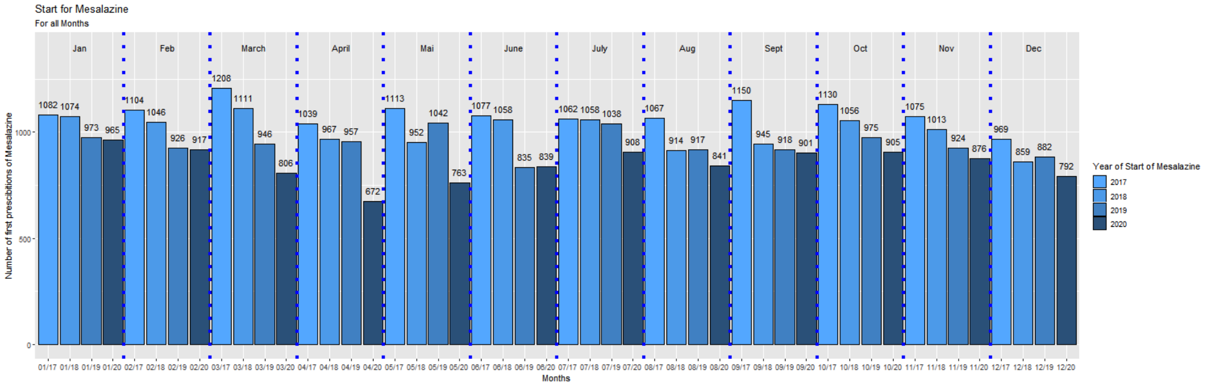

Supplemental Figure S3. Start of mesalazin (2017-2020).

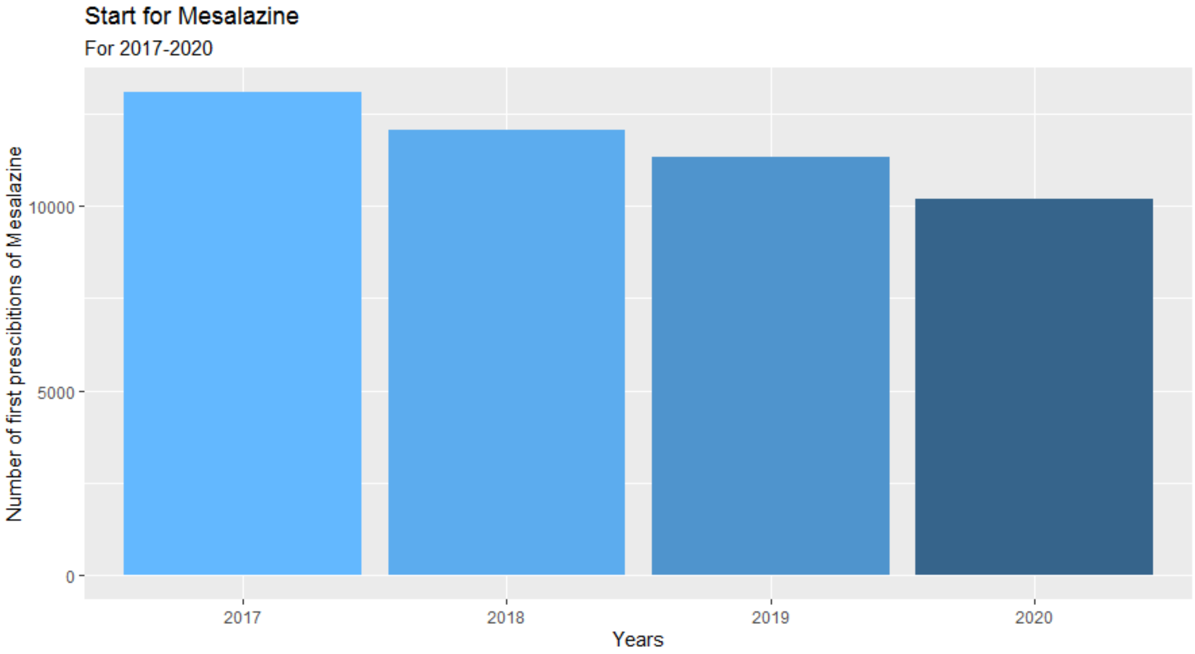

Supplemental Figure S4. Annual start of mesalazine from 2017 to 2020.
